# Do expectations of recovery improve risk assessment for people with whiplash-associated disorders? Secondary analysis of a prospective cohort study

**DOI:** 10.1186/s12891-022-05242-8

**Published:** 2022-04-27

**Authors:** Alexandra R. Griffin, Michele Sterling, Carrie Ritchie, Annette Kifley, Jagnoor Jagnoor, Ian D. Cameron, Trudy Rebbeck

**Affiliations:** 1 Faculty of Medicine and Health, The University of Sydney, D18 Susan Wakil Health Building, Western Ave, Camperdown, NSW 2050 Australia; 2 John Walsh Centre for Rehabilitation Research, Faculty and Medicine and Health, The University of Sydney, St Leonards, NSW Australia; 3 Recover Injury Research Centre, The University of Queensland, Herston, QLD Australia; 4 The George Institute for Global Health, The University of New South Wales, Kensington, NSW Australia

**Keywords:** Whiplash injuries, Evidence-based health care, Prognosis, Clinical decision rules, Cohort

## Abstract

**Background:**

WhipPredict, which includes prognostic factors of pain-related disability, age and hyperarousal symptoms, was developed and validated for prediction of outcome in people with whiplash associated disorders (WAD). Patient expectations of recovery was not an included factor, though is known to mediate outcomes. The aim of this study was to determine whether the addition of expectations of recovery could improve the accuracy of WhipPredict.

**Methods:**

Two hundred twenty-eight participants with acute WAD completed questionnaires (WhipPredict and expectations of recovery) at baseline. Health outcomes (neck disability index (NDI) and Global Perceived Recovery (GPR)) were assessed at 6- and 12-months post injury. Cut-off points for expectations of recovery predictive of both full recovery (NDI ≤10 % , GPR ≥ 4) and poor outcome (NDI ≥30 % , GPR ≤  − 3) were determined, and multivariate logistic regression analyses were used to compare models with and without this variable.

**Results:**

Expectations of recovery improved or maintained the accuracy of predictions of poor outcome (6-months: sensitivity 78 to 83%, specificity maintained at 79.5%; 12-months: sensitivity maintained at 80%, specificity 69 to 73%). The sensitivity of predictions of full recovery improved (6-months: 68 to 76%; 12-months: 57 to 81%), though specificity did not change appreciably at 6 months (80 to 81%) and declined at 12 (83 to 76%). ROC curves indicated a larger and more consistent improvement in model performance when expectations of recovery were added to the pathway predictive of full recovery.

**Conclusions:**

The addition of expectations of recovery may improve the accuracy of WhipPredict, though further validation is required.

**Supplementary Information:**

The online version contains supplementary material available at 10.1186/s12891-022-05242-8.

## Background

The management of whiplash-associated disorders (WAD) presents a considerable challenge to clinicians and researchers alike. Recovery is poor [1–3], associated with high personal and economic costs [4, 5], and clinical manifestations are diverse [6, 7]. Recent longitudinal studies have indicated that recovery, if it is to occur, will occur within the first 3 months of injury [1, 2, 8]. At one year following injury, approximately 50% of an inception cohort will have recovered, whilst 25% will continue to experience mild levels of pain and disability. The remaining 25% will experience more significant levels of pain and disability [1, 3, 9], often demonstrating minimal responsiveness to targeted interventions [10] and accumulating substantial personal and economic costs as a result [11]. For this reason, the early identification of individuals at risk of poor prognosis may be key in optimising outcomes and subsequently reducing the economic and societal burden of WAD.

Risk assessment tools may assist in the early identification of individuals who are more likely to have poor outcomes following injury. Risk assessment tools create pathways to aid decision-making by providing quantitative probabilities for prognosis, diagnosis or treatment effect based on specific patient characteristics or variables [12, 13]. There are three key stages of development each must undergo; derivation, validation and impact analysis. Each stage has important methodological considerations, for example, during derivation, data should be acquired from prospective longitudinal cohorts [12] of adequate size to accommodate 10–15 study participants per predictor variable [13, 14]. Whilst multiple risk assessment tools exist for estimating the likelihood of recovery following WAD [15–25], their statistical approaches vary considerably, as do their outcomes of interest. For example, Bohman et al. (2012) [17] developed a model for the prediction of recovery from WAD using seven variables; age, number of days to report the motor vehicle collision, headache before injury, pain other than neck and back, neck pain intensity, low back pain intensity and expectations of recovery. The primary outcome was global self-perceived recovery and concordance statistics revealed a c-index (or area under receiver operator curve; AU ROC, of 0.68, 95% CI: 0.65–0.71). Shortly following the publication of this study, the ‘Danish Whiplash Group Risk Assessment Score’ (DWGRAS) [16] was published, with an AU ROC of 0.79 for ‘total risk score’ in predicting 1-year work disability. The DWGRAS calculated a total risk score from three variables; neck pain and headache intensity scores (0–10, where 0 = no pain, 10 = worst imaginable pain, with the highest score of either neck pain or headache intensity considered), the total number of non-painful complaints (e.g. parasthesia, dizziness, fatigue; with 1 point allocated for the presence of each of 11 different complains), and total active neck mobility (combined active cervical range of motion into flexion, extension, right and left lateral flexion and right and left rotation). This score was then used to classify individuals into one of seven risk strata. The positive likelihood ratio (+LR) ranged from 1.0–7.0 across the strata.

In the same year, ‘WhipPredict’ (formerly the ‘Whiplash clinical prediction tool’) was derived [15]. WhipPredict is one of the few risk assessment tools for WAD that has undergone validation [26] and the only tool that has commenced impact analysis [27]. WhipPredict uses age, neck pain-related disability, and hyperarousal symptoms associated with posttraumatic stress symptoms to predict two distinct prognostic pathways; full recovery and chronic moderate/severe pain and disability. During the derivation of WhipPredict, a positive predictive value (PPV) of 71% was identified for full recovery in individuals with NDI scores of 32% or less, aged 35 years and under. The same PPV was identified for ongoing moderate to severe pain and disability in individuals with NDI scores of 40% or more, aged 35 years or older, and reporting hyperarousal symptoms scoring 6 or more on the hyperarousal subscale of the post-traumatic stress diagnostic scale (PDS). Subsequent validation work has since confirmed WhipPredict’s utility, with full recovery and chronic moderate to severe pain and disability pathways offering PPVs of 80 and 91% respectively [26]. In addition, WhipPredict has been found feasible for use by clinicians [26], perceived as user-friendly, fast and simple to apply in clinical settings. Given the accuracy of WhipPredict, together with its sound methodological underpinnings and unparalleled progress toward widespread implementation in WAD, opportunities to further optimise this tool are valuable. Since ‘recovery’ is a complex and highly patient-specific construct [28], inclusion of additional predictor variables that tap into these aspects of recovery may be one avenue to furthering the predictive utility of WhipPredict.

Patient expectations of recovery was not included in the derivation process for WhipPredict although this belief is known to influence outcomes in WAD [29, 30]. Previous work examining expectations of recovery in 6015 adults with WAD found that patients that expected to recover quickly improved 3 times faster than those that did not expect to recover [30]. Expectations of recovery relate to the belief that a particular health outcome will be achieved (self-efficacy), and are thought to be a product of individual’s prior health experiences and health literacy, together with the social and cultural contexts within which they exist [31]. In WAD, expectations of recovery have been identified to predict pain-related disability [8, 29, 30], neck pain intensity [30], and global perceived recovery [8, 30], and are endorsed in international clinical guidelines for WAD as an indicator of prognosis [32–34]. Though data assessing expectations of recovery were unavailable at the time of derivation of WhipPredict, the unique contribution of several other potential predictor variables for recovery in WAD were assessed during this process. These variables were selected on the basis of their role in the prediction of recovery, identified from previous reviews and cohort studies [15]. These included initial neck pain-related disability, cold pain threshold, age and posttraumatic stress symptoms [35], as well as initial neck pain intensity [1, 3, 36], gender [1], presence of headache and range of neck movement [37]. Recently, additional potential predictor variables for recovery from WAD have been identified, including expectations of recovery, the Short-Form 12 (SF-12) mental and physical component summary scores, the Euro-Qol 5-dimension 3-level quality of life questionnaire (EQ5D3L) and the therapeutic relationship [8]. However, of these variables, expectations of recovery was considered the only variable feasible for inclusion in WhipPredict. In this context, feasibility refers to the ease of application of the variable within WhipPredict, and relates to its complexity, completion time, interpretation, and cost.

A further and final consideration in the clinical utility of WhipPredict is its ability to predict more than one outcome. Recently, a core outcomes set (COS) for whiplash was developed [38]. A COS refers to an agreed set of outcome domains that have been endorsed by various clinical, research and industry stakeholders for inclusion in all clinical trials concerned with a specific clinical area or condition [39]. COS are effective in reducing outcome measure heterogeneity, thereby facilitating meaningful meta-analyses and promoting the development of a robust evidence base. Six outcome domains were identified within the whiplash COS, including physical functioning, perceived recovery, work and social functioning, pain severity, psychological functioning, and quality of life [38]. Given that multiple core domains have been implicated as essential in better understanding and managing WAD, prediction tools with the ability to predict more than one outcome are advantageous. Therefore, the present study had two key aims; our primary aim was to determine whether the addition of expectations of recovery to WhipPredict could improve its accuracy in predicting full recovery and/or poor outcome following WAD. Our secondary aim was to determine whether WhipPredict could predict outcomes other than neck pain-related disability.

## Methods

### Study design

This study was a secondary analysis of a prospective, multi-centre inception cohort study, known as the ‘FISH’ (Factors influencing social and health outcomes after land transport injury) study [40].

### Setting

Participants with acute WAD were recruited between 3rd November, 2013 and 17th May, 2016, from public hospital emergency departments, private physiotherapy practices and the State Insurance Regulatory Authority (SIRA) databases in New South Wales, Australia. This study was approved by the Sydney Local Health District Ethics Committee; reference number HREC/13/CRGH/67.

### Participants

**Fig. 1:**
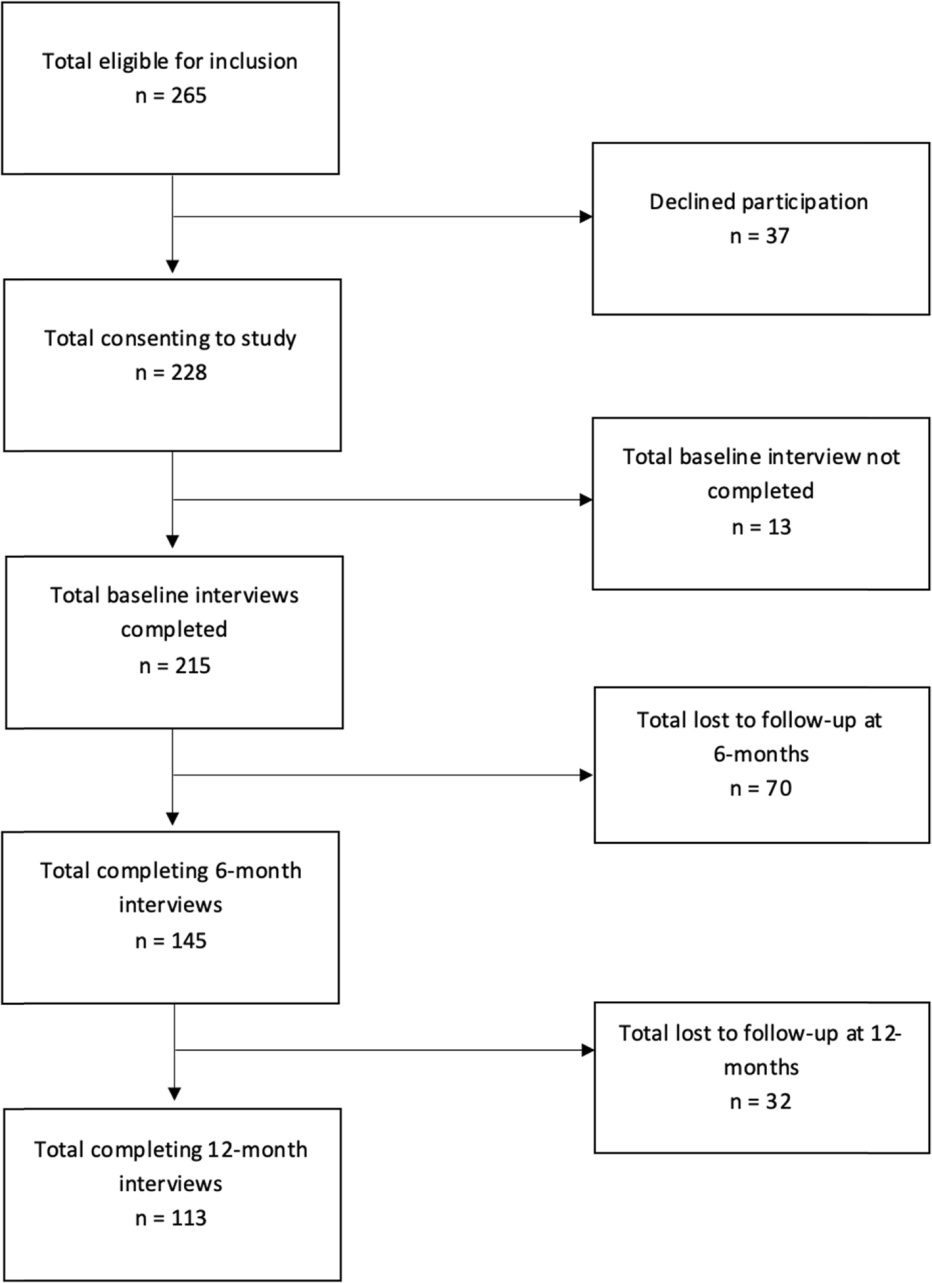
Flow of participants throughout the study

Participants with WAD were eligible for inclusion in they were aged > 17 years, reported neck pain following a motor vehicle crash consistent with WAD grade I-III [41] and were within 28 days of injury. Participants were excluded if they had suffered severe physical or psychological injury as a result of the motor vehicle crash (e.g., WAD IV, spinal cord injury, death of family member). Detailed inclusion and exclusion criteria are described elsewhere [40]. Participants completed a baseline questionnaire at recruitment and follow-up questionnaires at 6- and 12-months (Fig. 1).

### Baseline questionnaires

Baseline questionnaires collected demographic information including age, gender, recruitment source, educational level and comorbidities, where relevant (Table 1). Pain, disability and psychological measures were also collected at baseline. Average pain intensity over the past week was assessed using the numeric pain rating scale (NPRS) [42, 43] with subjective pain assessment ranging from 0/10 (no pain) to 10/10 (worst pain possible). Scores greater than 3/10 are suggestive of moderate to severe interference with functioning [44]. The Pain Catastrophising Scale (PCS; score range 0–52/52) [45] assessed pain catastrophising, with scores ≥ 25/52 indicative of clinically significant catastrophic thinking in relation to pain. Neck-specific pain-related disability was assessed using the Neck Disability Index (NDI) [46]. The NDI is the most frequently used outcome measure for assessing disability in WAD and has good reliability, construct validity and responsiveness [47]. Ten items with 6 possible scores (0–5; 0 = no disability, 5 = total disability) are summed to produce a total score out of 50, which may be multiplied by 2 to produce a percentage score. Scores ≤ 10% are considered to indicate full recovery, whilst scores ≥ 30% are considered to reflect ongoing moderate to severe disability. Expectations of recovery were assessed using item 7 of a modified short-form Orebro Musculoskeletal Pain Screening Questionnaire (SF-OMPSQ) [48–50]. Here, respondents were asked to rate the risk that their pain would become persistent: *‘On a scale of 0, “no risk”, to 10, “very large risk”, in your view, how large is the risk that your current pain may become persistent?’* Psychological measures assessed in baseline questionnaires included the revised Impact of Events Scale (IES-R) [51, 52], the Depression, Anxiety and Stress Scale (DASS) [53] and the hyperarousal subscale of the Posttraumatic Stress Diagnostic Scale (PDS) [54]. IES-R scores range from 0 to 88, with higher scores indicative of greater distress and predictive of increased risk of non-recovery. The PDS differs from the IES in that is has been mapped specifically against the Diagnostic and Statistical Manual of Mental Disorders (DSM-IV) diagnostic criteria for posttraumatic stress disorder. The hyperarousal subscale is one of three subscales of the PDS, scored using 5 items with scores ranging from 0 (not at all or only one time) to 3 (5 or more times a week/almost always) to provide a sum score between 0 and 15/15.

**Table 1:** Descriptive data

Variables	N	All participants (***n*** = 215)	Lost to follow-up at 12 m (***n*** = 102)	***P***-value	Predicted full recovery (***n*** = 35)	Predicted neither full recovery nor chronic moderate/severe pain and disability (***n*** = 106)	Predicted chronic moderate/severe pain and disability (***n*** = 66)	***P***-value
Age, mean (SD), in years	205	42 (16)	40 (15)	0.98	26 (7)	41 (16)	53 (12)	*< 0.001*
Gender, n (%)	198							
Male		89 (45)	33 (45)	0.24	11 (34)	44 (44)	30 (50)	0.35
Female		109 (55)	50 (55)		21 (66)	56 (56)	30 (50)	
Recruitment source, n (%)	204							
Hospital		177 (87)	74 (87)	0.16	29 (83)	90 (85)	57 (92)	0.79
Physiotherapist		8 (4)	5 (4)		2 (6)	5 (5)	1 (2)	
Insurance database		8 (4)	4 (4)		1 (3)	5 (5)	2 (3)	
Claims Advisory Service		11 (5)	8 (5)		3 (9)	6 (6)	2 (3)	
Education level, n (%)	204							
Pre-Primary		1 (0.5)	0 (0)	0.36	0 (0)	0 (0)	1 (2)	0.67
Primary		13 (6)	9 (6)		1 (3)	9 (9)	3 (5)	
Secondary		55 (27)	25 (27)		11 (31)	27 (26)	17 (28)	
Technical or any further education		56 (27.5)	24 (27.5)		7 (20)	29 (27)	19 (31)	
University of tertiary institution		79 (39)	33 (39)		16 (45)	41 (39)	22 (36)	
Comorbidities, n (%)	204							
Arthritis		45 (22)	16 (22)	0.17	0 (0)	29 (27)	16 (26)	*0.002*
Asthma		44 (22)	15 (22)	0.11	5 (14)	30 (68)	9 (15)	0.057
Anxiety		43 (21)	19 (21)	0.95	7 (20)	22 (21)	14 (23)	0.945
Degenerative disc disease		21 (10)	6 (10)	0.12	2 (6)	11 (10)	8 (13)	0.536
Depression		51 (25)	23 (25)	0.94	9 (18)	25 (49)	17 (27)	0.855
Diabetes		15 (7)	5 (7)	0.36	0 (0)	4 (4)	11 (18)	*< 0.001*
Obesity		23 (11)	9 (11)	0.58	5 (14)	11 (10)	7 (11)	0.819
Pain/Disability, mean (SD)								
NDI total score (0–50/50)	209	21 (9)	21 (10)	0.14	11 (4)	20 (9)	28 (5)	< 0.001**
Pain during past week (NRS: 0-10/10)	203	7 (2)	7 (2)	0.87	6 (2)	6 (2)	8 (2)	< 0.001**
PCS total score (0–52/52)	209	22 (14)	23 (14)	0.42	16 (10)	20 (13)	29 (13)	< 0.001***
SF-OMPQ expectations of recovery (0–10/10)	210	4 (3)	4 (3)	0.47	3 (2)	4 (3)	5 (3)	< 0.001***
Psychological								
IES-R total score (0–88/88)	210	39 (24)	41 (24)	0.33	34 (23)	33 (23)	52 (21)	< 0.001***
DASS total score (0–63/63)	211	20 (18)	21 (18)	0.95	14 (15)	17 (16)	28 (18)	< 0.001***
PDS hyperarousal (0–15/15)	204	8 (4)	8 (4)	0.83	6 (5)	7 (4)	10 (3)	< 0.001***

*Abbreviations*: *NDI* Neck disability index, *NRS* Numeric rating scale, *PCS* Pain catastrophising scale, *OMPSQ* Orebro Musculoskeletal Pain Questionnaire, *IES-R* Impact of Events Scale- revised, *PDS* Posttraumatic Stress Diagnostic scale, *DASS* Depression, Anxiety and Stress Scale. Post hoc significance: **predicted recovery vs neither vs chronic, ***predicted recovery and neither vs chronic

### Six and 12-month follow-up questionnaires

Follow-up questionnaires assessed two key outcomes at 6- and 12-months; pain-related disability measured by the NDI, and the 11-point Global Perceived Recovery (GPR) scale. The GPR scale requires patients to rate their improvement or decline in health status since a pre-determined time point. It is a simple, reliable tool that is easy to interpret and fast to administer [55]. The present study utilized an 11-point scale whereby patients rated perceived recovery from − 5 (vastly worse), through 0 (no change) to + 5 (greatly improved). Full recovery was defined as GPR scores ≥ 4 [8] and NDI scores ≤ 10% [15]. Poor recovery, hereafter referred to as *poor outcome*, was defined as GPR scores ≤ -3/5 and NDI scores ≥ 30% [15].

### Sample size

This study was a secondary analysis of data from a larger cohort study [40]. A sample size of at least 10 outcome events per predictor variable is recommended for the development of clinical prediction rules [13, 14]. Our sample contained 145 participants at 6 months and 113 participants at 12 months, and based on participant outcomes at these timepoints, was sufficient for exploring the accuracy of the current WhipPredict. Although our sample was small with respect to the additional analyses required to explore the addition of expectations of recovery, we decided a priori to pursue this question given its clinical significance and utility in informing future validation studies.

### Statistical analyses

Statistical analyses were performed using IBM SPSS Statistics version 27. Baseline variables of interest were summarized and presented for all participants, together with those lost to follow up at 12-months post injury. Distributions were assessed using the Shapiro Wilk test and between group comparisons were performed using either t-tests in the case of normally distributed data, or the Kruskal-Wallis non-parametric test for data that did not satisfy this assumption. Categorical data were assessed for associations using Chi-square analyses.

Univariate logistic regression analyses were performed to assess the association between expectations of recovery and recovery at 6- and 12-months for both NDI and GPR outcomes. Where *P* < 0.05, the addition of expectations of recovery to WhipPredict was considered, and further statistical analyses were undertaken. Firstly, appropriate cut-off points for expectations of recovery were established for both the ‘full-recovery’ and ‘chronic moderate/severe pain and disability’ pathways of WhipPredict. This was established for both NDI and GPR outcomes. For the full recovery pathway, subjects were dichotomized as either ‘fully recovered’ (NDI ≤ 10%; GPR ≥ 4) or as experiencing mild/moderate/severe disability (NDI > 10%; GPR < 4). For the chronic moderate/severe pain and disability pathway, subjects were dichotomized as having either chronic moderate/severe pain and disability (NDI ≥ 30%; GPR ≤ -3) or as being partially or fully recovered (NDI < 30%; GPR > − 3). Receiver operator characteristic (ROC) curves were created for both pathways, for both outcomes. The threshold for expectations of recovery scores above or below which the positive case would be expected to fall was calculated as the point of the curve nearest the upper left-hand corner and derived mathematically using the formula *d =* √ *(1- sensitivity)*^*2*^ *+ (1 – specificity)*^*2*^*.*

Next, the newly established cut points for expectations of recovery were used to compare the accuracy of the full recovery and chronic moderate/severe pain and disability pathways with and without the addition of expectations of recovery. Subjects were dichotomized into the two pre-established WhipPredict pathways, described above, and multivariate logistic regression analyses were used to compare the variance in outcome explained by each pathway (Nagelkerke R^2^) with and without expectations of recovery. Predicted probabilities from each regression analysis were saved and used to create ROC curves to determine an appropriate cut-off point above which the pathway-relevant ‘event’ (full recovery or poor outcome) would be expected. This threshold was calculated using the formula above for determining the point of the curve nearest the upper left-hand corner. Predicted probabilities were then dichotomized based on these newly derived cut-off points to compare proportions of expected versus observed positive cases for each pathway, per outcome. Accuracy statistics were used to compare pathways with and without the addition of expectations of recovery.

## Results

Two-hundred and twenty-eight individuals with acute WAD were eligible for inclusion in the present study and provided informed consent. Of these, 143 and 113 completed 6- and 12-month follow-up questionnaires respectively. Flow of participants through the trial is shown in Fig. 1.

**Fig. 2:**
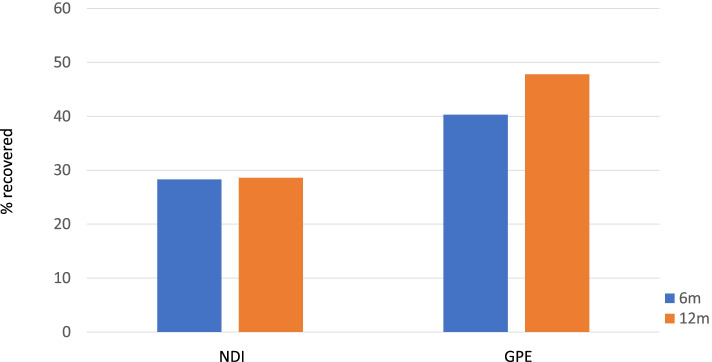
Percentage of cohort experiencing full recovery (NDI scores of ≤ 10%, GPR scores of ≥ 4) at 6- and 12-months, by outcome

Table 1 displays the baseline characteristics of the cohort and has been adapted from a similar table published elsewhere [8]. The majority (55%) were female, with a mean age of 42 years. Using WhipPredict, 35 (17%) individuals were predicted to experience full recovery, 66 (32%) chronic moderate/severe pain and disability, and 106 (51%) neither full recovery nor chronic pain and disability (Table 1). There were no significant differences in baseline characteristics amongst those lost to follow-up at 12 months. Individuals expected to experience full recovery according to WhipPredict had significantly lower levels of pain, disability and psychological distress than those expected to experience chronic moderate/severe pain and disability. Recovery, measured by NDI scores ≤ 10%, was observed in approximately one third of the cohort at 6 months (26 of 92 individuals, 28.3%; Fig. 2) and did not change appreciably by 12 months (22 of 77 individuals recovered; 28.6%). When recovery was assessed using GPR, 40.3% of the cohort had recovered at 6 months (58 of 144 individuals; Fig. 2), and this increased to 47.8% (54 of 113 individuals) at 12 months (Fig. 2).

Table 2 shows the results of the univariate logistic regression analyses performed to assess the association between expectations of recovery and recovery at 6- and 12-months for both NDI and GPR outcomes. Expectations of recovery were significantly associated with recovery at 6- and 12-months for both outcomes. This variable was subsequently carried forward into further analyses designed to test its unique contribution to the current WhipPredict model.

**Table 2:** Results of univariate logistic regression analyses for investigation of associations between expectations of recovery and recovery outcomes at 6- and 12-months

Outcome	Time-point	n	OR	95% CI	***P***-value	Nagelkerke R^**2**^
NDI	6-months	92	.693	.564–.853	0.001	.226
	12-months	77	.648	.507–.829	0.001	.266
GPR	6-months	145	.801	.706–.909	0.001	.119
	12-months	113	.808	.700–.933	0.004	.106

*Abbreviations*: *NDI* Neck disability index, *GPR* Global perceived effect

**Fig. 3:**
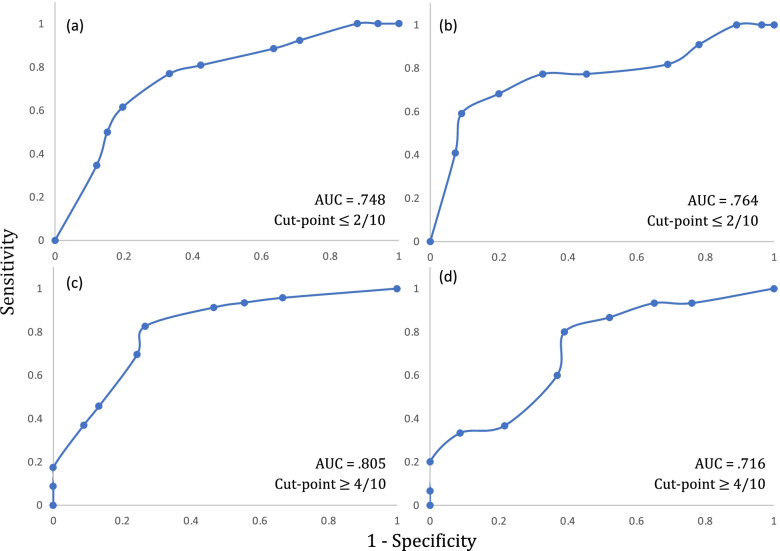
ROC curves used to determine cut-points for expectations of recovery score using NDI as the outcome at (**a**) 6-months for the recovery pathway, (**b**) 12-months for the recovery pathway, (**c**) 6-months for the chronic moderate/severe disability pathway, (**d**) 12-months for the chronic moderate/severe disability pathway

**Fig. 4:**
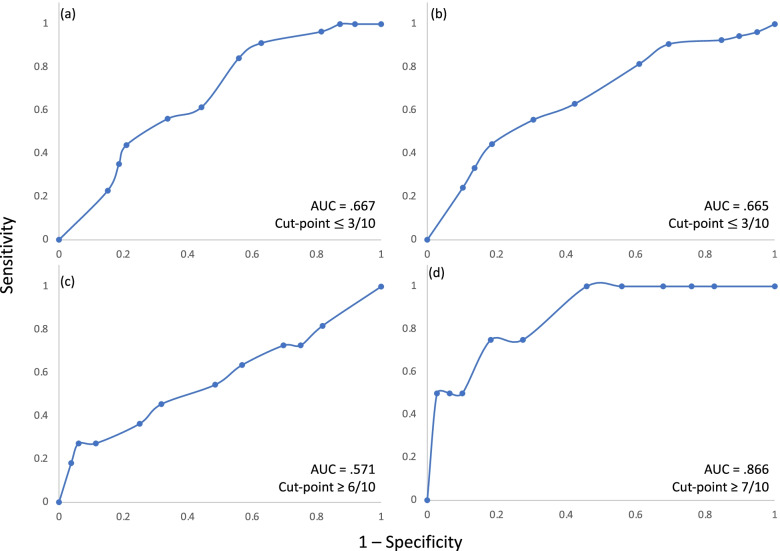
ROC curves used to determine cut-points for expectations of recovery score using GPR as the outcome at (**a**) 6-months for the recovery pathway, (**b**) 12-months for the recovery pathway, (**c**) 6-months for the non-recovery pathway, (**d**) 12-months for the non-recovery pathway

Using NDI as the outcome, ROC curve analyses revealed cut-off points for expectations of recovery at ≤ 2/10 for the full recovery pathway at 6- and 12-months, and ≥ 4/10 for the pathway predictive of poor outcome at 6- and 12-months (Fig. 3). Cut-off points were slightly greater for expectations of recovery when GPR was used as the outcome, with scores ≤ 3/10 predictive of full recovery, and scores ≥ 6/10 and ≥ 7/10 predictive of poor outcome at 6- and 12-months respectively (Fig. 4).

### Comparison of models with NDI as the outcome

**Fig. 5:**
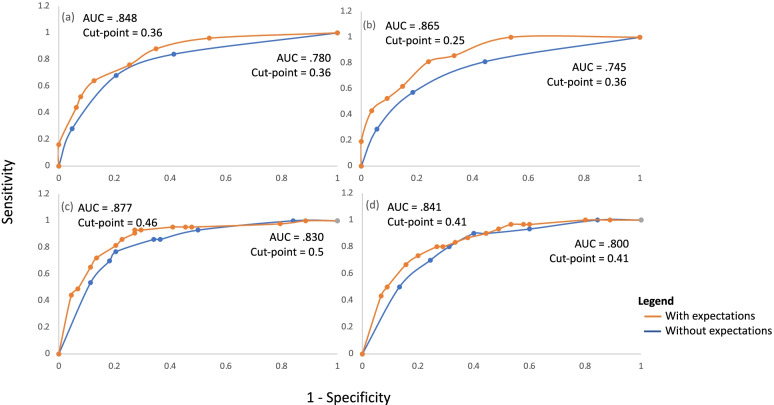
ROC curves for predicted probability of full recovery at (**a**) 6 months and (**b**) 12 months with and without the addition of expectations of recovery to the model. Also shown is the predicted probability of poor outcome at (**c**) 6 months and (**d**) 12 months with and without the addition of expectations of recovery to the model. The outcome of interest was NDI

Figure 5 presents the ROC curves produced from predicted probabilities of the ‘event’ (recovery or poor outcome) for NDI outcomes, generated from multivariate logistic regression analyses for full recovery (Fig. 4a and b) and poor outcome (Fig. 4c and d). Each plot compares predicted probabilities with and without the addition of expectations of recovery. Observed versus expected ‘event’ for each timepoint was used to generate the accuracy statistics for pathways at 6- and 12-months with and without the addition of expectations of recovery (Table 3). The addition of expectations of recovery to the WhipPredict pathway predictive of poor outcome resulted in modest improvements in most aspects of model performance at 6-months, where NDI was the outcome of interest. The exception to this was specificity, which was unchanged (79.5%) and -LR, which was very slightly improved (0.27 to 0.21). Closer inspection of accuracy statistics revealed the improvement observed in sensitivity was attributable to an increase in true positives (36 to 38) and subsequent reduction in false negatives (10 to 8). At 12-months, the specificity of the model was increased with the addition of expectations of recovery (69 to 73.3%). Sensitivity was unchanged and small improvements were observed in PPV (63.1 to 67%), NPV (83.7 to 84.6%), +LR (2.57 to 3), *χ*^*2*^ (17.21 to 20.51), AU ROC (.800 to .841) and Nagelkerke R^2^ (.364 to .438). These improvements were largely attributable to reductions in the false positive rate (14 to 12) and increases in the true negative rate (31 to 33).

**Table 3:** Accuracy statistics, area under receiver operator curves and Nagelkerke R^2^ for full recovery and chronic moderate/severe pain and disability pathways at 6- and 12-months. Values are shown for each pathway with and without the addition of expectations of recovery

	6-months				12-months			
	Full recovery		Chronic mod/severe disability		Full recovery		Chronic mod/severe disability	
NDI	Without expectations of recovery *n* = 91	With expectations of recovery *n* = 91	Without expectations of recovery *n* = 90	With expectations of recovery *n* = 90	Without expectations of recovery *n* = 74	With expectations of recovery *n* = 75	Without expectations of recovery *n* = 75	With expectations of recovery *n* = 75
True positive	17	19	36	38	12	17	24	24
False positive	13	12	9	9	9	13	14	12
False negative	8	6	10	8	9	4	6	6
True negative	53	54	35	35	44	41	31	33
Sensitivity (%)	68	76	78.2	82.6	57	81	80	80
Specificity (%)	80.3	81.8	79.5	79.5	83	76	69	73.3
PPV (%)	56.6	61.3	80	80.8	57.1	57	63.1	67
NPV (%)	86.8	90	77.7	81.4	83	91	83.7	84.6
+ LR	3.45	4.18	3.82	4.04	3.36	3.36	2.57	3
- LR	0.39	0.29	0.27	0.21	0.52	0.25	0.29	0.28
X^2^	19.14	26.99	30.06	34.82	11.94	20.4	17.21	20.51
AU ROC	.782	.837	.833	.887	.745	.865	.800	.841
R^2^	.299	.397	.438	.564	.229	.468	.364	.438
GPR	Without expectations of recovery *n* = 141	With expectations of recovery *n* = 140	Without expectations of recovery *n* = 141	With expectations of recovery *n* = 140	Without expectations of recovery *n* = 110	With expectations of recovery *n* = 110	Without expectations of recovery *n* = 110	With expectations of recovery *n* = 113
True positive	34	37	9	9	35	34	4	4
False positive	17	19	32	32	26	23	42	21
False negative	22	18	2	2	17	18	0	0
True negative	68	66	98	97	32	35	64	85
Sensitivity (%)	60.7	67.2	81.8	81.8	67.3	65.4	100	100
Specificity (%)	80	77.6	75.4	75.2	55.2	60.3	60	80.2
PPV (%)	66.6	66	21.9	21.9	57.4	59.6	8	16
NPV (%)	75.5	78.6	98	98	65.3	66	100	100
+ LR	3.03	3	3.32	3.3	1.5	1.65	2.5	5.05
- LR	0.49	0.42	0.24	0.24	0.59	0.57	0	0
X^2^	24.23	28	16	15.9	5.6	7.27	5.8	14.11
AU ROC	.713	.711	.803	.806	.628	.662	.844	.908
R^2^	.216	.212	.224	.223	.067	.107	.259	.336

*Abbreviations*: *NDI* Neck disability index, *GPR* Global perceived effect, *PPV* Positive predictive value, *NPV* Negative predictive value, *+LR* Positive likelihood ratio*, −LR* Negative likelihood ratio, *X*^*2*^ Chi squared statistics, *AU ROC* Area under receiver operator characteristic curve

Conversely, the effects of adding expectations of recovery to the pathway predictive of full recovery were mixed. At 6 months, most accuracy statistics improved, with sensitivity increasing (68 to 76%) along with markers of model fit (AU ROC .782 to .837; R^2^ .299 to .397). Specificity was maintained at 80%. However, at 12 months, whilst sensitivity increased substantially (57 to 81%), specificity reduced (83 to 76%). This decline was due to an increase in the number of false positives and a reduction in the detection of true negatives.

### Comparison of models with GPR as the outcome

**Fig. 6:**
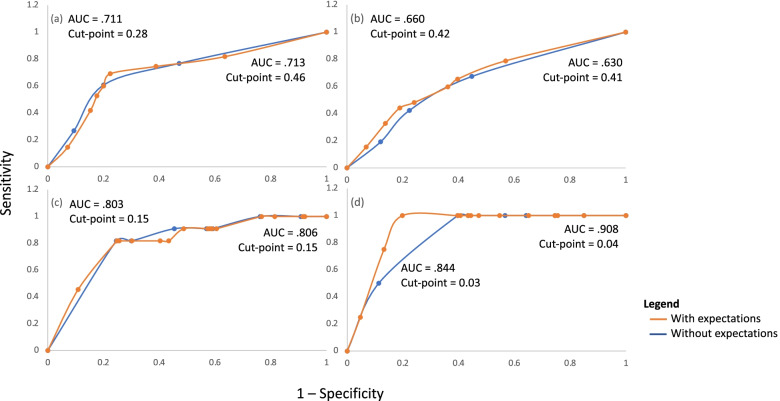
ROC curves for predicted probability of recovery at (**a**) 6 months and (**b**) 12 months with and without the addition of expectations of recovery to the model. Also shown is the predicted probability of poor outcome at (**c**) 6 months and (**d**) 12 months with and without the addition of expectations of recovery to the model. The outcome of interest was GPR

Figure 6 shows ROC curves for predicted probabilities of full recovery (Fig. 6a and b) and poor outcome (Fig. 6c and d), using GPR as the outcome. The accuracy statistics generated using the cut-off points identified from these curves are presented in Table 3. At 6-months, there was little change in the accuracy of the pathway predicting poor outcome when expectations of recovery was added. However, at 12 months, all variables increased (specificity 60 to 80%, PPV 8 to 16%, +LR 2.5 to 5, *χ*^*2*^ 5.8 to 14, AU ROC .844 to .908, Nagelkerke R^2^ .259 to .336), with the exception of sensitivity (100%) and NPV (100%) that were maximal. The 100% sensitivity rate is explained by the ratio of true positives (*n* = 4) to false negatives (*n* = 0), and the high NPV is explained by the ratio of false negatives (n = 0) to true negatives (*n* = 64 without expectations of recovery, and *n* = 85 with expectations of recovery).

Again, the addition of expectations of recovery produced mixed results when added to the recovery pathway. Specificity was reduced at 6 months (80 to 77.6%) whilst sensitivity was increased (60.7 to 67.2%). PPV remained similar (66.6 to 66%) and NPV increased (75.5 to 78.6%). The reverse was seen at 12 months, with sensitivity reducing slightly (67.3 to 65.4%) and specificity increasing (55.2 to 60.3%).

## Discussion

The addition of patient expectations of recovery to WhipPredict appeared to offer some benefit in improving the accuracy of the tool. We identified evidence supportive of the addition of this variable to both the pathway predictive of full recovery and the pathway predictive of poor outcome within WhipPredict. At present, WhipPredict, both with and without expectations of recovery, is not recommended for prediction of GPR given the high false positive rate observed.

The identification of individuals likely to experience poor outcome is a priority in the management of WAD, and on inspection of accuracy statistics, WhipPredict+E appeared to offer additional accuracy over WhipPredict for this purpose. Indicators of accuracy and model fit were either maintained or improved when WhipPredict+E was used to predict poor outcome, compared to WhipPredict, in the present cohort. At 6 months post injury, the sensitivity of WhipPredict+E was greater than WhipPredict, with small increases in the identification of true positives and reductions in false negatives. Sensitivity refers to the ability of a prediction tool or test to correctly identify individuals that will experience the predicted outcome [56]. It is closely linked to the ‘true positive’ rate and was higher than that seen in both the WhipPredict derivation and validation populations (43.5% in both populations compared to 78.2% with WhipPredict and 82.6% with WhipPredict+E; Additional file 1: Appendix A). We found that WhipPredict+E effectively re-classified two false negatives, identified by WhipPredict, into the pathway predictive of poor outcome, thereby increasing the true positive rate. The ability to correctly identify individuals that will experience poor outcomes is crucial to inform the early provision of targeted care and referral for specialist management as necessary. In addition, the accurate prediction of outcome at 6 months post injury is particularly relevant in New South Wales (NSW), Australia, where our cohort was located. Recent reforms to the NSW compensation scheme for individuals injured in motor vehicle crashes now provide funding for medical and rehabilitation services for a period of up to 6 months [57]. The provision of funded rehabilitation services after this time is possible, though not commonly provided. It is therefore valuable for clinicians to assess the likelihood of poor outcome at this timepoint and adjust treatment plans accordingly.

At 12 months post injury, sensitivity was maintained whilst specificity improved with WhipPredict+E. The specificity of a prediction tool refers to its ability to correctly identify, or rule out, individuals that will *not* experience the predicted outcome [56]. This is important in the prediction of poor outcome in WAD, where incorrect identification may result in unnecessary treatment and may impede natural recovery. Specificity was improved in WhipPredict+E with the re-classification of two false positive cases into true negatives. Comparative data for the specificity of this pathway across the WhipPredict derivation and validation populations at 12 months is not available, though recent work [49] using data from this cohort identified a pattern of overall lower specificity in WhipPredict compared to sensitivity (overall specificity 27.3%, overall sensitivity 92.7% at 12 months). Although the objectives of the present study warranted a pathway-specific approach to the investigation of accuracy, the evidently low *overall* specificity observed in Sterling et al. (2021) would suggest that any improvements in specificity, pathway-specific or otherwise, are beneficial. These findings should be validated in future studies.

The overall effect of adding expectations of recovery to the pathway predictive of full recovery was mixed. Whilst all accuracy parameters and markers of model fit improved at 6 months, this effect was less consistent at 12 months. The false positive rate was seen to increase, whilst the true negative rate declined marginally. This resulted in a reduction in specificity at 12 months from 83 to 76%. However, this was coupled with a relatively large increase in sensitivity (57 to 81%), driven by an increase in true positives and a reduction in false negatives.

A key consideration facing the authorship team was whether an improvement in the sensitivity or specificity of a prognostic pathway following the addition of expectations of recovery justified a reduction in its counterpart. Examination of ROC curves (Fig. 5) showed a consistently superior performance for the prognostic accuracy of WhipPredict+E relative to WhipPredict in the prediction of full recovery compared to the prediction of poor outcome. Given this, the role of expectations of recovery in improving the prediction of full recovery appears important and should be further investigated. It is possible that the mixed picture observed for WhipPredict+E in predicting full recovery may be due, in part, to the differences in cut points at which accuracy statistics were compared. For this reason, it is pertinent to consider accuracy statistics together with ROC curves to provide a balanced overview of the utility of this variable in improving predictive accuracy.

Adding to the evidence supportive of WhipPredict+E for the prediction of both full recovery and poor outcome were the changes observed in likelihood ratios (LR). LR are considered important accuracy statistics as they are independent of injury prevalence [56]. Whilst a more consistent pattern of improvement in LR was observed when WhipPredict+E was used to predict poor outcome, the +LR was greatest for predictions of full recovery at 6 months using WhipPredict+E. Here, the likelihood that someone classified into this pathway would go on to experience full recovery was over 4 times greater than those not classified into this pathway. Positive LRs ranged from 2.57–3.82 in WhipPredict and 3 to 4.18 in WhipPredict+E, supporting the clinical utility of WhipPredict+E. The improved performance of WhipPredict+E supports previous studies endorsing the prognostic utility of this variable for prediction of pain and disability outcomes and highlights the significance of future work to externally validate our findings.

The secondary aim of our study was to determine whether WhipPredict could predict outcomes other than neck pain-related disability. Following the recent development of a core outcomes set (COS) for WAD [38], six core domains were identified as essential in the management of WAD. Therefore, risk assessment tools that predict more than one outcome may be advantageous. In our cohort, WhipPredict was able to predict poor outcome with respect to GPR at 12 months with high sensitivity and specificity. Whilst the low (*n* = 0) false negative rate was an interesting finding from our cohort, and indicative that individuals *not* classified into this pathway could be ruled out from experiencing poor outcome, the high false positive rate was problematic. Using WhipPredict, 42 individuals (38%) were incorrectly classified as ‘positive’ for experiencing a poor outcome at 12 months. Although this rate dropped to 21 (19%) with the WhipPredict+E, this still constitutes a significant proportion of individuals that were incorrectly classified, as reflected in the very low PPV of 16%. The PPV refers to the probability that individuals classified into this pathway will, in fact, go on to experience the predicted poor outcome. It indicates the proportion of individuals correctly classified into the pathway (‘true positives’) of all those classified (‘positives’), both true and false [56]. Although the pathway has captured all true positives (*n* = 4), it has captured a significant proportion of false positives, resulting in a very low PPV. We therefore consider WhipPredict (and WhipPredict+E) inappropriate for predicting poor global recovery. Though WhipPredict performed better in the prediction of full recovery, its reduced ability to accurately predict poor outcome would likely render its use confusing for both clinicians and researchers. Our findings support the recommendation that since risk assessment tools such as WhipPredict are derived to predict outcomes specific to a chosen outcome measure, in this case the NDI, their use is not usually generalizable to other measures [58]. With this in mind, the derivation of a tool designed specifically to predict global recovery is necessary, particularly in view of the significance of this construct with respect to the patient-specific nature and complexity of perceived recovery [28].

The findings of this study must be considered in relation to its limitations. This study was a secondary analysis of a subset of data from a larger study. The addition of the NDI was requested specifically for this subset of individuals, and a delay in the addition of this variable to the follow-up questionnaires resulted in some missing data for this variable. Despite this limitation, there were several strengths of the current study. We followed participants over a 12-month period, with our multi-centre recruitment design including an inception cohort (recruitment was within one month of injury). This facilitated data acquisition from a large number of participants across NSW, Australia. The collection of expectations of recovery data at baseline additionally facilitated exploration of this variable within WhipPredict for the first time, adding to the current knowledge base.

Whilst our study has established that WhipPredict and WhipPredict+E are not suitable tools to predict patient perceived global recovery, we identified preliminary evidence that WhipPredict+E may offer increased accuracy over WhipPredict in identifying those likely to experience both poor outcome and full recovery. This opportunity is significant in the context of improving outcomes following WAD. The benefits seen in the prognostic accuracy of WhipPredict+E are important and should be validated in an external cohort.

## Supplementary Information

**Additional file 1: Appendix A**. Comparison of accuracy statistics for the likelihood of chronic moderate/severe pain and disability for the derivation, validation, and current study populations at 6-months post injury.

## Data Availability

The data from this study is part of a larger study, the results of which are available elsewhere. For this reason, data from this study will not be shared. The author for correspondence may be contacted for queries specific to the data of this study.

## Abbreviations

COS: Core outcomes set
DWGRAS: Danish Whiplash Group Risk Assessment Score
DASS: Depression, anxiety, stress scale
EQ5D3L: Euro-Qol 5-dimension 3- level quality of life questionnaire
FISH: Factors influencing social and health outcomes
IES: Impact of events scale
LR: Likelihood ratio
NDI: Neck disability index
NSW: New South Wales
NPRS: Numeric pain rating scale
OMPSQ: Orebro musculoskeletal pain screening questionnaire
PCS: Pain catastrophizing scale
PPV: Positive predictive value
PDS: Posttraumatic stress diagnostic scale
ROC: Receiver operator characteristic
SF-12: Short-form 12
SIRA: State Insurance Regulatory Authority
WAD: Whiplash associated disorder
