# Supplementary material for: Do expectations of recovery improve risk assessment for people with whiplash-associated disorders? Secondary analysis of a prospective cohort study
Source: BMC Musculoskelet Disord. 2022 Apr 27;23:395. doi: 10.1186/s12891-022-05242-8 (PMC9044895; doi:10.1186/s12891-022-05242-8)
Supplement: Supplementary file 1 — Additional file 1: Appendix A. Comparison of accuracy statistics for the likelihood of chronic moderate/severe pain and disability for the derivation, validation, and current study populations at 6-months post injury. [file 12891_2022_5242_MOESM1_ESM.docx]

**Appendix A**. Comparison of accuracy statistics for the likelihood of chronic moderate/severe pain and disability for the derivation, validation, and current study populations at 6-months post injury.

|  | Derivation population^15^ | Validation population^26^ | Current population | Current population WhipPredict+E |
| --- | --- | --- | --- | --- |
| Sensitivity | 43.5% | 43.5% | 78.2% | 82.6% |
| Specificity | 93.8% | 98.7% | 79.5% | 79.5% |
| Positive likelihood ratio | 7 | 33.9 | 3.82 | 4.04 |
| Negative likelihood ratio | 0.6 | 0.6 | 0.27 | 0.21 |
| Positive predictive value | 71.4% | 90.9% | 80% | 80.8% |
